# Histone deacetylase inhibitiors enhance sensitivity of murine sarcoma tumors to IL-13-PE immunotoxin-based cancer therapy by upregulating IL-13Rα2 expression *in vitro* and *in vivo*

**DOI:** 10.1186/2051-1426-1-S1-P73

**Published:** 2013-11-07

**Authors:** Toshio Fujisawa, Hideyuki Nakashima, Bharat H Joshi, Raj K Puri

**Affiliations:** 1 DCGT, OCTGT, CBER, US FDA, Bethesda, MD, USA; 2 NTT Medical Center Tokyo, Tokyo, Japan

## 

Interleukin-13 Receptor alpha 2 (IL-13Rα2) is a tumor antigen and a potent target for cancer therapy. Previously, we have reported that histone deacetylase (HDAC) inhibitors upregulated the IL-13Rα2 expression and enhanced anti-cancer effects of IL-13-PE, an immunotoxin composed of interleukin-13 and truncated *Pseudomonas* exotoxin, in a mouse model of human pancreatic cancer. Herein, we have investigated whether HDAC inhibitors can enhance the effect of IL-13-PE in immunocompetent mouse tumor models by upregulating IL-13Rα2. We analyzed mRNA and protein levels of mouse IL-13Rα2 in four mouse tumor cell lines, MCA304 sarcoma, 4T1 breast cancer, GL261 glioma and D5 melanoma. By RT-PCR analysis, mRNA levels for IL-13Rα2 were high in MCA304, moderate in 4T1, low in GL261 and below the detection limit in D5 mouse tumor cell lines. These cell lines were treated with three types of HDAC inhibitors, trichostatin A, sodium butyrate and suberoylanilide hydroxamic acid (SAHA) to evaluate their effects on IL-13Rα2 expression *in vitro*. All HDAC inhibitors dramatically increased IL-13Rα2 mRNA expression in MCA304, 4T1, and GL261 cell lines. The D5 tumor cell line showed only a slight increase in mRNA levels of IL-13Rα2. Western blot analysis for IL-13Rα2 demonstrated increased levels of IL-13Rα2 protein in all of the HDAC inhibitor treated tumor cells. We also observed that all three HDAC inhibitors selectively enhanced cytotoxicity of IL-13-PE in MCA304 and 4T1 cell lines. TSA treatment resulted in maximum improvement of IL-13-PE induced cytotoxicity in MCA304, NaB in GL261, and SAHA in 4T1 cell lines. We next developed a subcutaneous tumor model of mouse sarcoma by implanting MCA304 tumor cells in C57BL/6 mice. Mice were treated with SAHA (an FDA licensed drug) i.p. at 25mg/kg/day dose from day 4 through 9 prior to IL-13-PE immunotoxin treatment. IL-13-PE was administered at a 250mg/kg dose intratumorally from day 5 through 9. Mice treated with vehicle control and SAHA showed tumor growth to ~1500 mm^3^ in ~22 days and were sacrificed because of ethical reasons. Treatment with IL-13-PE alone showed a significant reduction in tumor growth and mice survived longer. Tumor size reached ~750 mm^3^ in 35 days. However, when IL-13-PE was administered to SAHA pretreated mice, tumor size showed further reduction and reached only ~400 mm^3^ on day 35. Additional studies are ongoing to determine the mechanism of synergistic antitumor effects of HDAC inhibitors and IL-13-PE. These results suggest that HDAC inhibitors may upregulate tumor antigens *in vivo* and thus may be considered in combination with cancer vaccines and immunotherapy products for cancer therapy.

